# Anaemia and low birth weight in Medani, Hospital Sudan

**DOI:** 10.1186/1756-0500-3-181

**Published:** 2010-06-28

**Authors:** Elhassan M Elhassan, Ameer O Abbaker, Abderahuim D Haggaz, Magid S Abubaker, Ishag Adam

**Affiliations:** 1 Department of Obstetrics and Gynecology, Faculty of Medicine, University of Geizera, P.O. Box 20, Wad Medani, Sudan; 2 Department of Obstetrics and Gynecology, Faculty of Medicine, University of Khartoum, P.O. Box 102, Khartoum, Sudan

## Abstract

**Background:**

Reducing the incidence of Low birth weight (LBW) neonates by at least one third between 2000 and 2010 is one of the major goals of the United Nations resolution "A World Fit for Children". This was a case-control study conducted between August-October 2009 in Medani Hospital, Sudan to investigate the risk factors for LBW. Cases were mothers who delivered singleton baby < 2500 gm. Controls were mothers delivered singleton baby of ≥ 2500 gm.

**Findings:**

Out of 1224 deliveries, 97 (12.6%) of the neonates were LBW deliveries. While maternal socio-demographic characteristics (age, parity and mother education) and anthropometrics measurements were not associated with LBW, lack of antenatal care (OR = 5.9, 95% CI = 1.4-24.4; *P *= 0.01) and maternal anaemia (OR = 9.0, 95% CI = 3.4-23.8; *P *< 0.001) were the main risk factor for LBW.

**Conclusion:**

Thus, more care on antenatal care and nutrition may prevent LBW.

## Introduction

Low birth weight (LBW) is an important indicator of obstetric care and health status. It continues to remain a major public health problem worldwide especially in the developing countries. LBW is an important determinant of child-hood morbidity, associated with death during infancy [1,2]. Reducing the incidence of LBW neonates by at least one third between 2000 and 2010 is one of the major goals of the United Nations resolution "A World Fit for Children" and is an important contribution toward Millennium Development Goal (MDG) 4 of reducing child mortality by two thirds by 2015 [3]. Thus, local surveillance and basic epidemiology can more accurately assess epidemiology of LBW; identify areas to which interventions should be targeted, and monitor the effectiveness of these interventions over time. An obvious example like preventing deaths of LBW babies requires the use of technologically advanced treatment methods associated with neonatal intensive care that is not feasible for poor populations. Therefore interventions could focus on decreasing LBW presumptively by improving maternal care and preventing the causes. Local surveillance and basic epidemiology are fundamental, so as to assess LBW and identify areas to which future interventions should be targeted. The aims of the present study were to investigate prevalence and the risk factors for LBW Medani Hospital, Sudan so as to add to our ongoing researches on maternal and perinatal epidemiology in this setting and anaemia and its effects among pregnant Sudanese women [4-9].

## Materials and methods

**Figure 1:**
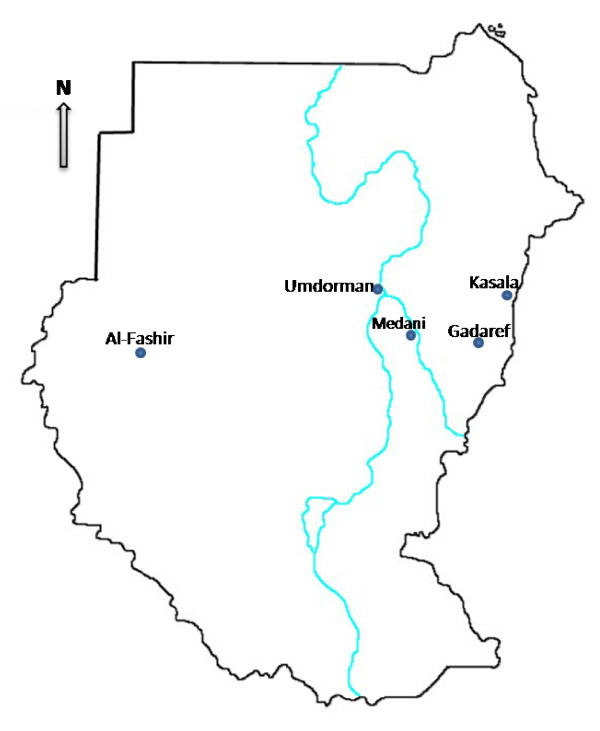
**Different regions of Sudan**.

This was a case-control study conducted between August-October 2009 in the labour ward of Medani Hospital in Central Sudan, figure 1. After obtaining an informed consent, women with a singleton neonate were approached to participate in the study. Those women with diabetes mellitus, hypertensive disorder of pregnancy, antepartum haemorrhage, renal disease, congenital malformed baby or any other medical problem were excluded. Case was a woman who delivered a baby weighted less than 2500 gm. For every case the subsequent woman who delivered a baby weighting ≥ 2500 gm acted as control. A structured questionnaire was administered to each woman to gather socio-demographic informations such as education, age, parity, and antenatal care attendance. Information on the first day of the last menstrual period before the index pregnancy and on the date of the previous pregnancy outcome (delivery and miscarriage) was gathered. The interpregnancy interval was defined as the time between the woman's previous delivery, miscarriage and the first day of the last menstrual period for the index pregnancy. The date of the last normal menstrual period was used to determine gestational age. However, when the discrepancy between gestational age determined in this way and gestational age calculated from ultrasound scanning was greater than 2 week, the ultrasound estimate was preferred. Maternal weight, height, and body mass index (BMI, calculated as weight in kilograms divided by height in meters squared) were obtained. Maternal hemoglobin was measured using HemoCue haemoglobinometer (HemoCue AB, Angelhom, Sweden). Maternal, placental and cord thick blood films were prepared. The slides were Giemsa stained and read counting the number of asexual Plasmodium *falciparum *parasites per 200 white blood cells and double checked blindly by an expert microscopist.

Preterm delivery was defined as labour before completed 37 weeks gestational age. Maternal anaemia was considered if her haemoglobin was < 11 g/dl. Anaemia was classified as mild (Hb: 9-10.9 gm/dl), moderate (7-8.9 gm/dl and severe anaemia (Hb: < 7 gm/dl), respectively. Neonates were weighed immediately to the nearest 50 gm and LBW considered if birth weight was less than 2500 gm.

## Statistics

Data were entered into a computer database and were double-checked before analysis using SPSS version 13.0 (SPSS, Chicago, IL, USA). Means and proportions for the socio-demographic characteristics were compared between the 2 groups of the study using the *t *test and χ^2 ^test, respectively. Univariate and multivariate analyses were performed. LBW was the dependent variable, while socio-demographic characteristics and medical and obstetrics events were independent variables. *P *< 0.05 was considered significant. When there was discrepancy between the results of the t test, χ^2 ^test, and the results of multivariate, the results of the multivariate analysis were taken as final.

### Ethics

The study received ethical clearance from the Research Board at the Faculty of Medicine, University of Khartoum, Sudan.

## Results

Out of 1224 singleton deliveries, 97 (12.6%) of the neonates were LBW deliveries. only 7 (7.2%) out of these 97 LBW deliveries were preterm deliveries and the rest were small for gestational age. The age and education were not different between cases and controls. In comparison with the controls, significantly more women in the case group were primipare [31 (31.9%) vs. 16(16.4%), *P *= 0.01] and did not attend antenatal care [29 (29.8%) vs. 14(14.4%), *P *= 0.001] in the index pregnancy. The mean (SD) of the maternal weight, height and BMI were significantly lower in the cases than in the controls. Significantly more women in case group had anaemia, 65 (67.0%) vs. 27(27.8%), *P *= 0.001, Table 1. While anaemia was moderate in 19 (29.2%), mild in 46 (70.8%) out of the 65 anaemic women in the case group, it was severe in one (3.7%), moderate in 2 (7.4%), and mild in 24 (88.8%) out of the 27 anaemic women in the control group.

**Table 1:** Comparing socio-demographic and obstetrical characteristics between cases and controls in Medani hospital, Sudan

Variable	Cases (*N *= 97)	controls (*N *= 97)	*P*. Value
Age, years	28.6(5.7)	29.3(6.2)	0.4
Primiparae	31 (31.9)	16(16.4)	0.01
Education < secondary level	36(37.1)	30(30.9)	0.6
Lack of antenatal care	29 (29.8)	14(14.4)	0.001
Maternal weight, kg	63.0 (7.8)	70.8 (13.5)	0.001
Maternal height, cm	162.4 (6.8)	164.7(7.2)	0.02
Body mass index	23.8(2.1)	25.9(4.0)	0.001
Anaemia	65 (67.0)	27(27.8)	0.001

Data were shown as mean (SD) or n (%) as applicable

Ten and 8 women in the case and control group, respectively had self reported malaria in the index pregnancy; *P *> 0.05. The blood films for *P. falciparum *malaria were positive in three sets of maternal, placental and cord *P. falciparum *malaria, 2 of these were in the cases and the rest (one) in the controls. One woman in the case group had placental positive blood films for malaria.

## Predictors for LBW

While maternal socio-demographic characteristics and anthropometrics measurement were not associated with LBW, lack of antenatal care (OR = 5.9, 95% CI = 1.4-24.4; *P *= 0.01) and maternal anaemia (OR = 9.0, 95% CI = 3.4-23.8; *P *< 0.001) were the main risk factor for LBW in multivariate analyses, table 2. Primiparous status (OR = 2.4, 95% CI = 1.2-4.8; *P *= 0.01) and maternal weight (OR = 2.1, 95% CI = 1.1-3.7; *P *= 0.01) were the risk factors for LBW in univariate analyses only, table 2.

**Table 2:** Factors associated with low birth weight in Medani Hospital, Sudan using univariate and multivariate analyses

Variable	Univariate analyses			Multivariate analyses		
	**OR**	**95% CI**	***P *value**	**OR**	**95% CI**	***P *value**

Maternal age	0.9	0.9-1.0	0.3	0.9	0.9-1.0	0.9
Primiparous status	2.4	1.2-4.8	0.01	2.7	0.3-27.7	0.3
Education < secondary	0.8	0.5-1.3	0.4	2.6	0.9-8.0	0.07
Lack of antenatal care	5.4	2.3-12.9	0.001	5.9	1.4-24.4	0.01
Interpregnancy interval	1.5	0.9-2.4	0.6	0.9	0.9-1.0	0.4
Maternal weight	2.1	1.1-3.7	0.01	0.7	0.3-1.0	0.5
Maternal height	1.0	0.5-1.7	0.9	1.1	0.5-2.4	0.6
Maternal BMI	0.9	9.0-1.0	0.9	1.7	0.1-20.0	0.6
Maternal anaemia	9.0	4.5-18.0	0.001	9.0	3.4-23.8	< 0.001

## Discussion

The main findings of the current study were; high rate of LBW, there was no significant association between maternal socio-demographic characteristics and anthropometrics measurement and LBW, anaemia was the main risk factor for LBW. The rate (12.6%) of LBW in this study was high like the rate of LBW that we have previously reported in Khartoum and eastern Sudan [8,10] and was higher than the rate (8.3%) of LBW reported by Elshibly and Schmalisch in Khartoum, Sudan [11].

Unlike the previous reports from eastern and central Sudan [8,10,12], maternal socio-demographic characteristics and anthropometrics measurement were not found to be risk factors for LBW in western Sudan [6] as well as in this study. However, even in this study maternal weight, height, BMI were significantly lower in those women who delivered LBW babies and maternal weight was the risk factor for LBW in univariate analysis only. Thus, these anthropometrics measurements could be just confounding factors. However it is necessary to point to the limitation of using anthropometric measurements taken during pregnancy to estimate the risk for LBW. Unlike measurements before pregnancy, these measurements are liable to changes; unfortunately pre-pregnancy measurements can seldom be taken in Africa, where women commonly present to health facilities only when they are advanced in pregnancy.

The current study showed that anaemic women were at nine times higher risk to deliver LBW babies. This goes with the previous observations from eastern and western Sudan as well as other African countries [6,8,13], where anaemia was reported to be a predictor of LBW and poor perinatal outcome. Anaemia during pregnancy is a big burden in Sudan where pregnant Sudanese women are more susceptible to anaemia regardless to their age and parity [7,9,14]. Moreover anaemia has been reported to be associated with fetal anaemia and still birth in eastern Sudan [8,15]. Only few women had malaria in this study. Due to fund constrain, blood film was the diagnostic tool used in the current study. Blood-smears for malaria detection may underestimate malaria in pregnant women. Placental histology, which is the gold standard was used in the before in the same hospital and placental malaria was the risk factor for LBW [16].

Like our previous reports in Khartoum [10], in the current study women who did not attend antenatal care were at six times higher risk of LBW. Antenatal care is one of the most effective ways of reducing maternal, perinatal mortality and morbidity, and under use has been associated with adverse maternal and perinatal outcomes [17]. Our recent reports have suggested that the high maternal and perinatal mortality rates in Western Sudan could be reduced by increasing women's use of antenatal care services [4,5].

In other African countries, maternal characteristics such as marital status, age gravidity and substance abuse have been reported to be associated with prematurity [18]. Smoking and the use of alcohol and other substances are not common among Sudanese women and all women who delivered are married. These factors have been not investigated (difficult to investigate) as confounders in our study.

Recently, in other countries other infectious diseases namely tuberculosis and periodontal diseases were the main risk factor for low birth weight in Taiwan and in Madagascar, respectively [19,20]. Paternal characteristics including age, height, and birth weight were associated with LBW [21]. Unintended, unwanted, and mistimed pregnancies ending in a live birth were associated with a significantly increased risk of LBW [22]. In India a recent study revealed that pre pregnancy maternal weight (< 45 kgs), anaemia in pregnancy and maternal age less than 20 years were the significant risk factors of low birth weight of term babies [23]. In Bangladesh, maternal age, educational level, antenatal care and economic status play an important role in the incidence of low birth weight [24].

In summary, this is small sample size hospital based study and may not represent what is going on in the community. This point (small sample size) may account for failure to show association between birth weight and the traditional factors like maternal age, parity, weight and height. Thus, more care on mother (maternal) nutrition and prevention of anaemia may prevent LBW in this setting.

## Competing interests

The authors declare that they have no competing interests.

## Authors' contributions

EME and IA carried out the study and participated in the statistical analysis and procedures. ADH, MSA and AOA coordinated and participated in the design of the study, statistical analysis and the drafting of the manuscript. All the authors read and approved the final version.
